# Pharmacological target sites for restoration of age‐associated deficits in NMDA receptor‐mediated norepinephrine release in brain

**DOI:** 10.1111/jnc.16280

**Published:** 2024-12-10

**Authors:** Yousef Aljohani, William Payne, Robert P. Yasuda, Thao Olson, Kenneth J. Kellar, Ghazaul Dezfuli

**Affiliations:** ^1^ Department of Pharmacology and Physiology Georgetown University Medical Center Washington District of Columbia USA

**Keywords:** aging, alpha‐2‐adrenergic receptors, NMDA receptors, norepinephrine release

## Abstract

Aging affects virtually all organs of the body, but perhaps it has the most profound effects on the brain and its neurotransmitter systems, which influence a wide range of crucial functions, such as attention, focus, mood, neuroendocrine and autonomic functions, and sleep cycles. All of these essential functions, as well as fundamental cognitive processes such as memory, recall, and processing speed, utilize neuronal circuits that depend on neurotransmitter signaling between neurons. Glutamate (Glu), the main excitatory neurotransmitter in the CNS, is involved in most neuronal excitatory functions, including release of the neurotransmitter norepinephrine (NE). Previous studies from our lab demonstrated that the age‐associated decline in Glu‐stimulated NE release in rat cerebral cortex and hippocampus mediated by NMDA glutamate receptors, as well as deficits in dendritic spines, and cognitive functions are fully rescued by the CNS stimulant amphetamine. Here we further investigated Glu‐stimulated NE release in the cerebral cortex to identify additional novel target sites for restoration of Glu‐stimulated NE release. We found that blockade of alpha‐2 adrenergic receptors fully restores Glu‐stimulated NE release to the levels of young controls. In addition, we investigated the density and responsiveness of NMDA receptors as a potential underlying neuronal mechanism that could account for the observed age‐associated decline in Glu‐stimulated NE release. In the basal state of the receptor (no added glutamate and glycine) the density of NMDA receptors in the cortex from young and aged rats was similar. However, in contrast, in the presence of 10 μM added glutamate, which opens the receptor channel and increases the number of available [^3^H]‐MK‐801 binding sites within the channel, the density of [^3^H]‐MK‐801 binding sites was significantly less in the cortex from aged rats.

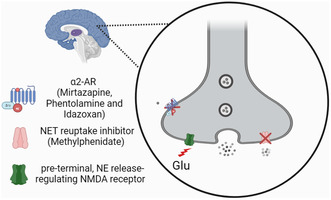

## Graphical abstract

Brain aging is characterized by a decrease in N‐methyl D‐aspartate mediated glutamate‐stimulated release of norepinephrine in the cerebral cortex. Proposed target sites for pharmacological restoration of deficits in norepinephrine release in the aging brain include blockade of alpha‐2 adrenergic receptors using mirtazapine or idazoxan, and targeting the norepinephrine reuptake inhibitor with the CNS stimulant methylphenidate.

**Abbreviations**
CNS: Central nervous system
Glu: Glutamate
MPH: Methylphenidate
NE: Norepinephrine
NMDA: N‐methyl‐D‐aspartic acid
TTX: Tetrodotoxin

## 1 INTRODUCTION

Aging is associated with alterations in CNS neurotransmitter systems (Lee & Kim, 2022). In particular, decreases in NE release and uptake activities, receptor expression, and signaling pathways have been found in the aging rat brain (Gargano et al., 2023; Gonzales et al., 1991; Pittaluga et al., 1993; Scarpace & Abrass, 1988). The cerebral cortex and hippocampus are vulnerable to age‐related changes (Scognamiglio et al., 2024). Moreover, the effects of the excitatory neurotransmitter glutamate (Glu) on NE neurotransmission may be altered in the aging brain (Abdallah et al., 2016; Navarro et al., 1994).

We previously showed that Glu‐stimulated NE release is significantly decreased in cerebral cortex and hippocampus brain slices from aging rats and that this release is mediated primarily by NMDA receptors (Scognamiglio et al., 2024). Those studies thus confirmed earlier studies that found a similar decrease in NE release stimulated by NMDA itself (Gonzales et al., 1991; Pittaluga & Raiteri, 1992).

In those studies, we demonstrated that this decreased release can be restored by the addition of the psychostimulant amphetamine to the slice incubation (Scognamiglio et al., 2024).

Moreover, we found that a two‐week treatment of aged rats with amphetamine increased the number of dendritic spines and particularly the number of mushroom‐shaped spines, which are thought to strengthen synapses and be more stable. Furthermore, this chronic amphetamine treatment improved performance on memory tasks in aged rats through its actions to augment NE neurotransmission at β‐adrenergic receptors (Scognamiglio et al., 2024). Here we further investigated Glu‐stimulated NE release in the cerebral cortex from young and aged rats to identify additional neuronal mechanisms and targets that can augment NE release, further assess the contribution of NMDA receptors in Glu‐stimulated NE release, and measure the density of these receptors and their regulation by glutamate in cortex from young and aged rats.

## 2 MATERIALS AND METHODS

### 2.1 Experimental animal model

Male Fischer 344 rats (18–22 months old) were obtained from the NIA breeding facility at Charles River Laboratories (USA), while young male Fischer 344 rats (2–4 months old) were procured from Charles River Laboratories (USA). Female Fischer 344 rats (18–22 months old) and young female Fischer 344 controls (2–4 months old) were used in a subset of experiments where indicated. All rats were group‐housed and maintained on a 12‐h light–dark cycle with ad libitum access to food and water. All experiments were approved by the Georgetown University Animal Care and Use Committee (Ref. No. 2017‐0008) and complied with NIH ethical guidelines.

### 2.2 Drugs and chemicals

[^3^H]‐NE (11.8 Ci/mmol; Part # NET377250UC) and [^3^H]‐MK‐801 (29.5 Ci/mmol; Part # NET 972250UC) with specific activity of 11.8 and 29.5 Ci/mmol, respectively, were purchased from Revvity; Waltham, MA, USA, L‐threo‐Methylphenidate hydrochloride (Cat # M6935) were purchased from Sigma‐Aldrich, Tetrodotoxin citrate (TTX; Cat # HB1035) was from Hello Bio, while other chemicals including L‐glutamic acid (Cat # G1251), NMDA (Cat # 01‐145‐0), phentolamine (Cat # 64‐311‐00), mirtazapine (Cat # 20‐185‐0), clonidine (Cat # 06‐901‐00), and idazoxan (Cat # AC473371000) were purchased from Thermo Fischer Scientific.

### 2.3 Norepinephrine release experiments

**FIGURE 1:**
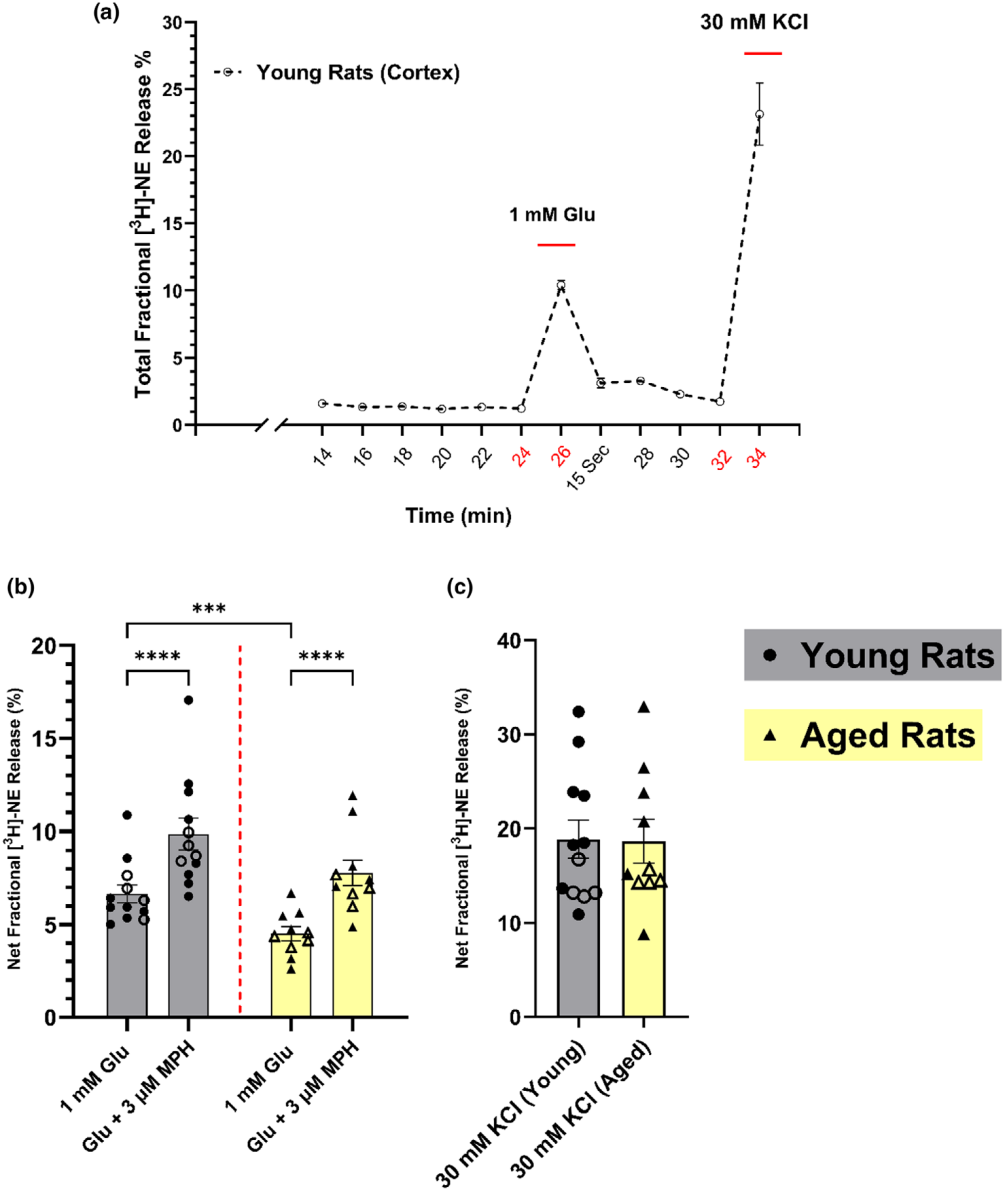
The effect of aging on [^3^H]‐NE release from young (2–3 months old) and aged (18–24 months old) Fischer 344 rat cortical brain slices and its rescue. In (a), the descriptive experimental timeline indicates total fractional NE release from young rat cortical slices (*n* = 7) in response to a 1 mM Glu stimulation (at 24–26 min time points) and 30 mM K^+^ (at 32–34 min time points). In (b), the age‐associated decline in the Glu‐stimulated [^3^H]‐NE release was rescued using 3 μM MPH in the cerebral cortex tissue slices from male (solid symbol) and female (open symbol) F344 rats (young rats *n* = 12 and aged rats *n* = 10). (c) 30 mM K^+^‐ evoked [^3^H]‐NE release in young versus aged rats. Each bar is the mean ± SEM of net fractional release (%) in each group after subtracting the basal fractional release (~1.49% of the total tritium content). Data were analyzed using two‐way ANOVA with Tukey's multiple comparison tests: ***p = 0.001, ****p < 0.0001, whereas the effect of aging on K^+^‐evoked release was analyzed using an unpaired *t*‐test. NE, norepinephrine; Glu, glutamate; KCL, potassium chloride; MPH, methylphenidate.

The NE release assay was conducted as previously described (Scognamiglio et al., 2024). The animals were euthanized using an overdose of isoflurane (Cat # NDC 59399‐106‐01; each mL containing 99.9% isoflurane) as per the guidelines from the Georgetown University's IACUC protocol for the euthanasia of research animals. In brief, animals are placed in a bell jar and exposed to gauze saturated with liquid isoflurane to create deep anesthesia (as confirmed by the lack of a response to a firm toe pinch) and then decapitated. The whole cortex was rapidly dissected and sliced crosswise at 200‐μm thickness with a McIlwain tissue chopper (The Mickie Laboratory Engineering Co., Gomshall, England). The tissue slices were gently dispersed in 10 mL oxygenated (95% O2/5% CO_2_) Krebs buffer (118 mM NaCl, 5 mM KCl, 2 mM KH_2_PO_4_, 24 mM NaHPO_3_, 2.5 mM CaCI_2_,11 mM D‐glucose, 0.2 mM L‐ascorbic acid, 25 mM HEPES (pH 7.4)), and allowed to settle before the buffer was aspirated. The tissue was resuspended with 10 mL of fresh Krebs buffer and mixed for 5 min on a rotator at room temperature. The buffer was aspirated again and the tissue slices were incubated with 2 mL of 100 nM [^3^H]‐NE in a shaking water bath at 37°C for 25 min. The tissue slices were then washed twice for 5 min with 10 mL Krebs buffer each to remove the extracellular [^3^H]‐NE and resuspended in Krebs buffer to a final concentration of 1 mg/μL. The assay was conducted in a set of 12‐well tissue culture plates. 20 μL aliquots of standard tissue mini slices (approximately 20 mg of tissue) were placed into nylon mesh baskets. The tissue baskets were then incubated and manually moved sequentially in wells containing 3 mL of buffer for six 2‐min intervals until a steady basal release of [^3^H]‐NE was sustained before stimulation (see Figure 1a). In the case of testing a drug effect on stimulus‐evoked release, the drug was added to the three sequential wells preceding stimulation. After stimulation, the tissue was briefly rinsed for 15 s to remove any residual effect of stimulus followed by incubation in 3 wells to reestablish basal release. The tissue was then incubated in Krebs buffer containing high potassium (30 mM) to stimulate to a near‐maximum [^3^H]‐NE release and to ensure tissue viability. The tissue was then lysed in 0.1 N NaOH to assess the remaining [^3^H]‐NE. The released [^3^H]‐NE in each well was quantified using a Beckman‐Coulter LS6500 Scintillation Counter. Total fractional [^3^H]‐NE release was calculated as the amount of released [^3^H]‐NE released in each well relative to the total [^3^H]‐NE in the tissue at that time point whereas net fractional release was determined by subtracting the mean of three basal release wells from the release observed under stimulus conditions.

### 2.4 [ ^3^H]‐MK‐801 binding

#### 2.4.1 Membrane preparation

The MK‐801 binding experiment was done as previously described (Reynolds 2001), with minor modifications. Briefly, frozen cortex tissues from Fischer 344 rats were homogenized in 20 mL of ice‐cold 10 mM HEPES buffer (pH 7.4) containing 1 mM EDTA. The homogenate was then washed 4 times by centrifugation at 34 000 *g* for 10 min at 4°C in buffer. The pellet was then resuspended in 20 mL of HEPES buffer, without EDTA, and incubated for 15 min at 37°C in a shaking water bath. The homogenate was centrifuged again and the final tissue pellet was resuspended in HEPES buffer without EDTA.

#### 2.4.2 Incubation and filtration

Aliquots of homogenates equivalent to 2 mg original tissue weight were added to test tubes containing 10 nM [^3^H]‐MK 801 in an assay volume of 0.5 mL. Non‐specific binding was measured in the presence of 100 μM unlabeled MK‐801. Specific binding was defined as the difference between total binding and non‐specific binding. Saturation bindings experiments were conducted with increasing concentrations of [^3^H]‐MK 801 (0.2–30 nM) to determine the affinity of the receptor (*K*_d_ = dissociation constant) and B‐max (total number of receptor binding sites). Receptor regulation by glutamate and glycine (Cat # J62407.36) was measured in the absence (basal binding condition) and presence of 10 μM Glu and 10 μM glycine (stimulated binding condition), these concentrations were chosen based on preliminary studies that showed they are sufficient to produce maximum binding of the [^3^H]‐MK 801. The tubes were incubated for 2 h at room temperature on an Orbital Shaker and then filtered through Whatman GF/C filters on a Brinkman Cell Harvester and counted in a Beckman Scintillation Counter (LS6500; Jersey City, NJ).

### 2.5 SDS‐PAGE and western blot

Tissue homogenates in 10 mM HEPES buffer were lysed in denaturing buffer (SDS). The denatured tissue lysates were separated on 7.5% acrylamide gels by SDS‐PAGE and then transferred to Immobilon‐FL PVDF membranes (Cat # 88518). A total protein stain was detected by the Total Revert 700 kit (LI‐COR Biosciences; Cat # *P*/*N 926‐11010*). Total protein normalization was used as an internal loading control (Bettencourt et al., 2020). Membranes were blocked using the LI‐COR Intercept Odyssey blocking buffer (Cat # *P*/*N 927‐70001*) diluted in PBST (50:50). The NMDA receptor NR2A and NR2B subunit polyclonal antibodies were used alongside the NR1 monoclonal antibody (PhosphoSolutions, CO, USA; Cat # 1805‐NR1). The NR1 monoclonal antibody was specifically developed and validated in our laboratory (Luo et al., 1997; Vicini et al., 1998; Wang et al., 1995). NMDA subunits were probed by primary antibodies with 25 μg of rabbit anti‐NR2B and 30 μg of mouse monoclonal anti‐NR1 and anti‐NR2A in a blocking solution overnight at 4°C. Bound antibodies were detected using a secondary antibody diluted (1/30 000) of anti‐mouse and anti‐rabbit in a blocking solution. Bands for the NMDA subunits were imaged simultaneously from the same sample lane on a single membrane using dual color detection. Densitometric analysis of Western blots was quantified using Odyssey Infrared Imaging System (LI‐COR Biosciences Model # 9120, Lincoln, NE). Sample proteins were quantified using the BCA Protein Assay Kit (Cat # A55860; Thermo Fisher Scientific).

### 2.6 Data analysis

2‐way ANOVA or mixed‐effects models were employed when more than two groups were compared and followed by the appropriate post hoc multiple comparisons test. An unpaired two‐tailed student *t*‐test was used to compare only two groups. An alpha significance level was set at *p* < 0.05 and ROUT testing was performed to identify outliers. EC_50_ and *E*_max_ values were obtained by fitting the values to a sigmoidal concentrations‐response curve with a variable slope identical to the three‐parameter logistic equation (Top, Bottom, and Log EC_50_). For all in vitro experiments, group sizes were estimated based on a priori power analyses (using G*power (Faul et al., 2007)), power at 0.8 (Beta =0.2), alpha at 0.05, and utilizing previous studies of a similar nature (Scognamiglio et al., 2024). Normality was assessed using a Kolmogorov–Smirnov test which indicated no significant deviation from normality (*p* > 0.05) allowing us to proceed with parametric analyses. Data were analyzed and plotted using GraphPad Prism 9 (RRID:SCR_002798) and Microsoft Excel software (RRID:SCR_016137).

## 3 RESULTS

### 3.1 Age‐related decline in Glu‐stimulated release of [ ^3^H]‐NE from the cortex of aged rats and its restoration by methylphenidate

We first conducted a series of validation experiments using a highly sensitive in vitro neurotransmitter release assay to optimize the experiment timeline and the stimulation period that produces an optimal signal‐to‐noise (basal release) ratio. As shown in the timeline of the [^3^H]‐NE release assay used here (Figure 1a), after 6 consecutive 2‐min baseline release periods, basal fractional [^3^H]‐NE release from these slices was stable, and the addition of 1 mM glutamate then stimulated release to approximately 7‐times the basal level. After re‐establishing basal release, the tissues were exposed to buffer containing a high concentration of potassium (30 mM) to measure near‐maximum [^3^H]‐NE release and to demonstrate tissue viability.

We previously found a decrease in Glu‐stimulated [^3^H]‐NE release mediated predominantly by NMDA receptors in cerebral cortical and hippocampal slices from aged rats (Scognamiglio et al., 2024), which is consistent with earlier studies that measured NE release in aged rat cortical and hippocampal slices stimulated by NMDA itself (Gonzales et al., 1991; Pittaluga & Raiteri, 1992). In addition, we demonstrated that the decreased release of [^3^H]‐NE in the brains from aged rats can be rescued by the addition of amphetamine (Scognamiglio et al., 2024). Here we again measured Glu‐stimulated release of [^3^H]‐NE in cortical slices from aged and young control rats to determine the potential restorative effect of methylphenidate on the age‐associated deficit in Glu‐stimulated [^3^H]‐NE release in the cortex of aged Fischer 344 rats. As shown in Figure 1b,c, a two‐way ANOVA revealed a significant main effect of age [*F*_(11,29)_ = 7.85; *p* < 0.0001] with aged rats displaying decreased NE release in comparison to young controls. This is consistent with our previous studies (Scognamiglio et al., 2024). Furthermore, a significant main effect of drug treatment [*F*_(3,29)_ = 37.35; *p* < 0.0001] was also observed. Follow‐up Tukey post hoc comparisons revealed that 3 μM methylphenidate significantly potentiates Glu‐stimulated [^3^H]‐NE release in the cortical slices from aged rats in comparison to control aged tissue slices (*p* < 0.0001). Importantly, methylphenidate restored Glu‐stimulated [^3^H]‐NE release in the cortical slices from aged rats to the levels seen in slices from the young control rats (Figure 1b). In contrast to NE release stimulated by glutamate, NE release stimulated by 30 mM K^+^ did not differ significantly in the cortex from young and aged rats (Figure 1c).

### 3.2 Inhibitory α2‐adrenoceptors as novel target sites for restoring Glu‐stimulated [ ^3^H]‐NE release in the aged brain

**FIGURE 2:**
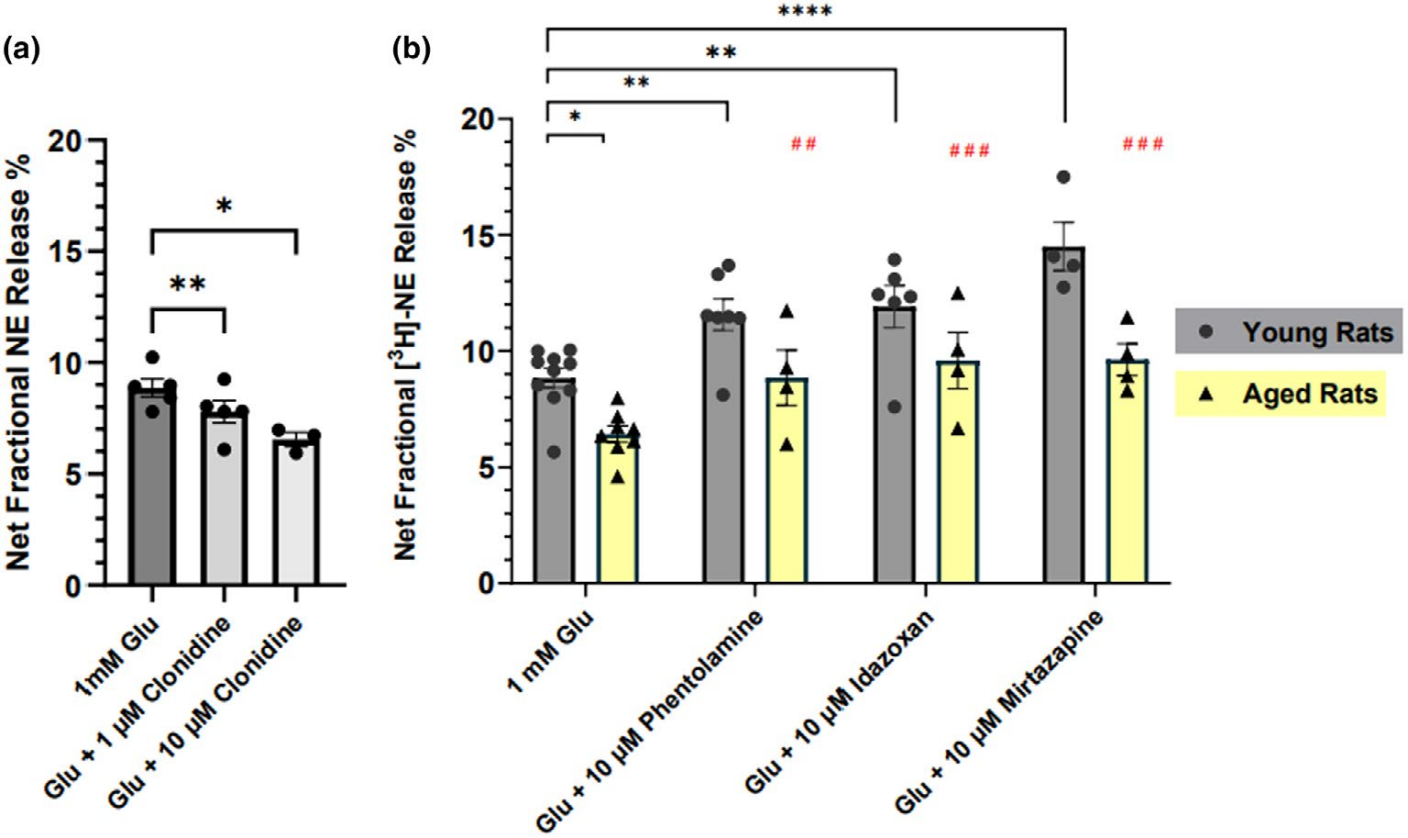
Presynaptic α2‐adrenoceptors receptors modulate the glutamate‐stimulated release of [^3^H] ‐NE in aged Fischer 344 rat cortical brain slices. In (a), α2‐adrenoreceptors receptors agonist, Clonidine, decreases glutamate (Glu)‐stimulated [^3^H]‐NE release in a concentration‐dependent manner in young rat cortex (*n* = 3–5). In (b), [^3^H]‐NE release was potentiated by antagonizing α2‐adrenoceptors receptors in the cerebral cortex from young (*n* = 4–11) and aged (*n* = 4–8) with 10 μM phentolamine, idazoxan, and mirtazapine. Each data point represents a duplicate of one animal. Data were analyzed using two‐way ANOVA with Dunnett's multiple comparison test; *p ≤ 0.05, **p ≤ 0.01, ****p < 0.0001; ^##^ *p* < 0.01, ^###^ *p* < 0.001 (# for aged rats comparison using a mixed‐effect analysis followed by Dunnett's multiple comparison test).

To determine whether treatments other than psychostimulants can augment Glu‐stimulated [^3^H]‐NE release, and particularly whether such treatments can restore the decreased release in the brains from aged rats, we investigated the possible role of inhibitory α2‐adrenoceptors in the cortex. These receptors modulate the release of NE in the autonomic nervous system (Langer et al., 1980; Starke; Weikop et al., 2004), as well as in rat brain (Carter, 1997; Garcia et al., 2004; Sacchetti et al., 2001). To determine if these inhibitory receptors are present in our cortical slice preparation, we first measured the effect of the α2‐adrenoceptor agonist clonidine on Glu‐stimulated [^3^H]‐NE release in young rats. Results from a mixed model ANOVA revealed a significant main effect of drug treatment [*F*_(1.408,4.223)_ = 36.76; *p* = 0.0022]; (Figure 2a). Specifically, follow‐up Dunnett's post hoc analyses demonstrate that clonidine dose‐dependently decreases Glu‐stimulated [^3^H]‐NE release in the cortex in our system (*p* = 0.0069; *p* = 0.0377 respectively). Thus, results with clonidine confirm these receptors are present and functional in the cortical slices used here. We next assessed the effects of blocking these receptors with phentolamine, which blocks both α1‐ and α2‐adrenoceptors; idazoxan, a selective α2‐adrenoceptor blocker; and mirtazapine, a clinically used tetracyclic antidepressant drug that acts primarily by blocking presynaptic α2‐adrenoceptors (Pinder, 2005), on the Glu‐stimulated [^3^H]‐NE release. As shown in Figure 2b, a two‐way ANOVA revealed a significant main effect of drug treatment with α2 blockers potentiating Glu‐stimulated [^3^H]‐NE release [*F*_(7,29)_ = 13.25; *p* < 0.0001]. Furthermore, post hoc comparisons using Dunnett's test indicated that idazoxan, phentolamine, and mirtazapine restored Glu‐stimulated [^3^H]‐NE release in cortical slices from the aged rats to approximately the levels seen in non‐treated control slices from the young rats (*p* > 0.05; Figure 2). Thus, these α2‐adrenoceptor blockers are as effective as methylphenidate in augmenting NE release in the aged rat brain. Consistent with this mechanism of action, a separate analysis was done to investigate the effect of drugs on aged rats only. A mixed‐effect analysis followed by Dunnett's multiple comparison test showed a significant effect of drug treatment on NE release in cortical slices from aged rats [*F*_(3,9)_ = 32; *p* < 0.0001]. Thus, in aged rats, both phentolamine and idazoxan potentiate the decrease in the Glu‐stimulated release of [^3^H]‐NE in the cortex (*p* = 0.0013; *p* = 0.0002, respectively). Moreover, mirtazapine, similar to the effects of phentolamine and idazoxan, increased the Glu‐stimulated release of [^3^H]‐NE in aged brains (*p* = 0.0002), and restored the decrease in the Glu‐stimulated release of [^3^H]‐NE in cortex from aged rats to the level seen in young rats. The effect of these α2‐adrenoceptor antagonists to augment the release of NE indicates the presence of active α2‐adrenoceptors in the rat cerebral cortex. Although our studies do not specifically address the location of these receptors, they are likely presynaptic release‐modulating auto receptors on NE axon terminals. In any case, the present data suggest that blockade of α2‐adrenoceptors may represent a useful pharmacological target site to augment NE release, with possible clinical implications for conditions associated with impaired NE release in the brain, including aging.

### 3.3 Presynaptic Glu‐stimulated NE release‐regulating NMDA receptor target sites in the cortex are TTX‐sensitive

**FIGURE 3:**
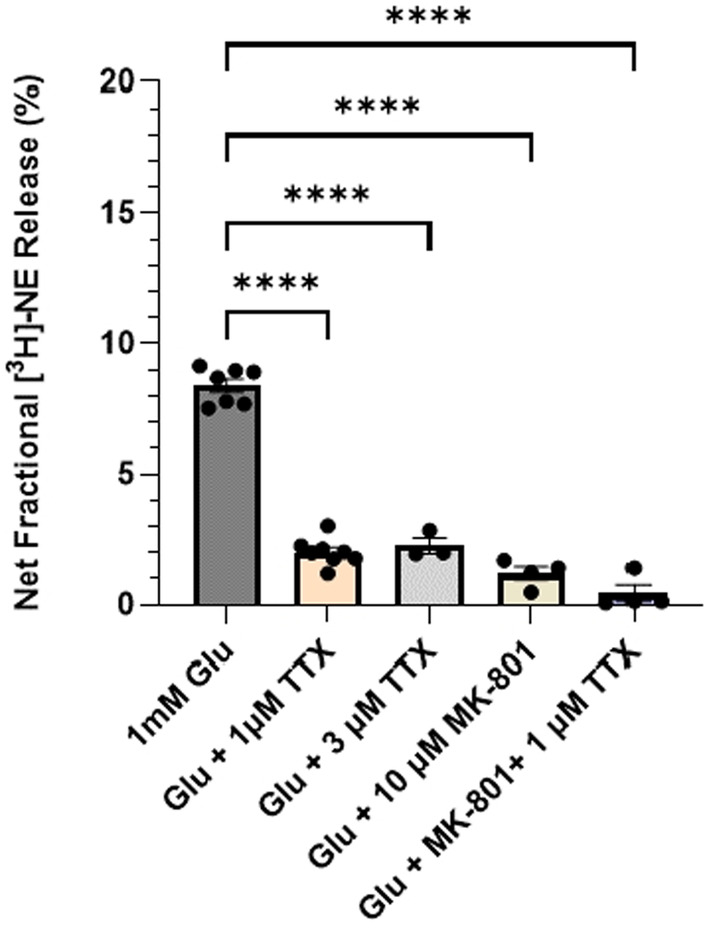
Characterizing the cellular localization of the NMDA receptors regulating [^3^H] ‐NE release in young rat cortical brain slices. 1 mM Glu‐stimulated‐[^3^H]‐NE releases in the cerebral cortex tissue slices from young rats (*n* = 3–7) in the presence 1 & 3 μM of the TTX, voltage‐gated Na channel blocker, 10 μM MK‐801, and a combination of MK‐801 and TTX. Data are expressed as mean (±SEM) of net fractional release (stimulated—basal), with each data point representing a duplicate from one animal. Data were analyzed using a mixed‐effect analysis followed by Dunnett's multiple comparison test ****p < 0.0001. NE, norepinephrine; Glu, glutamate; TTX, tetrodotoxin.

We have previously shown that Glu‐stimulated NE release in the rat cerebral cortex is calcium‐dependent and mediated primarily by NMDA receptors (Scognamiglio et al., 2024). To determine if this Glu‐stimulated NE release is dependent on one or more synaptic circuits, we blocked synaptic transmission with tetrodotoxin (TTX), which inhibits action potentials by blocking voltage‐gated sodium channels. As shown in Figure 3, a mixed‐effect analysis followed by Dunnett's multiple comparison test revealed a significant effect of treatment on Glu‐stimulated NE release in rat cortical tissue slices [*F*_(4,14)_ = 242.2; *p* < 0.0001]. Thus, 1 μM TTX decreased Glu‐stimulated NE release by 75% (*p* < 0.0001) and there was no further decrease when the TTX concentration was increased to 3 μM, indicating this was the maximum inhibitory effect of TTX. As a comparison, we measured Glu‐stimulated NE release in the presence of the NMDA receptor channel blocker MK‐801(10 μM), which blocked ~88 percent of the release (*p* < 0.0001; Figure 3). Interestingly, the combination of MK‐801 with TTX effectively eliminated Glu‐stimulated release (*p* < 0.0001), suggesting that the release remaining after TTX alone is probably mediated by NMDA receptors, possibly located on NE axons.

### 3.4 NMDA is a potent high‐affinity, partial agonist compared to glutamate in mediating the release of norepinephrine

**FIGURE 4:**
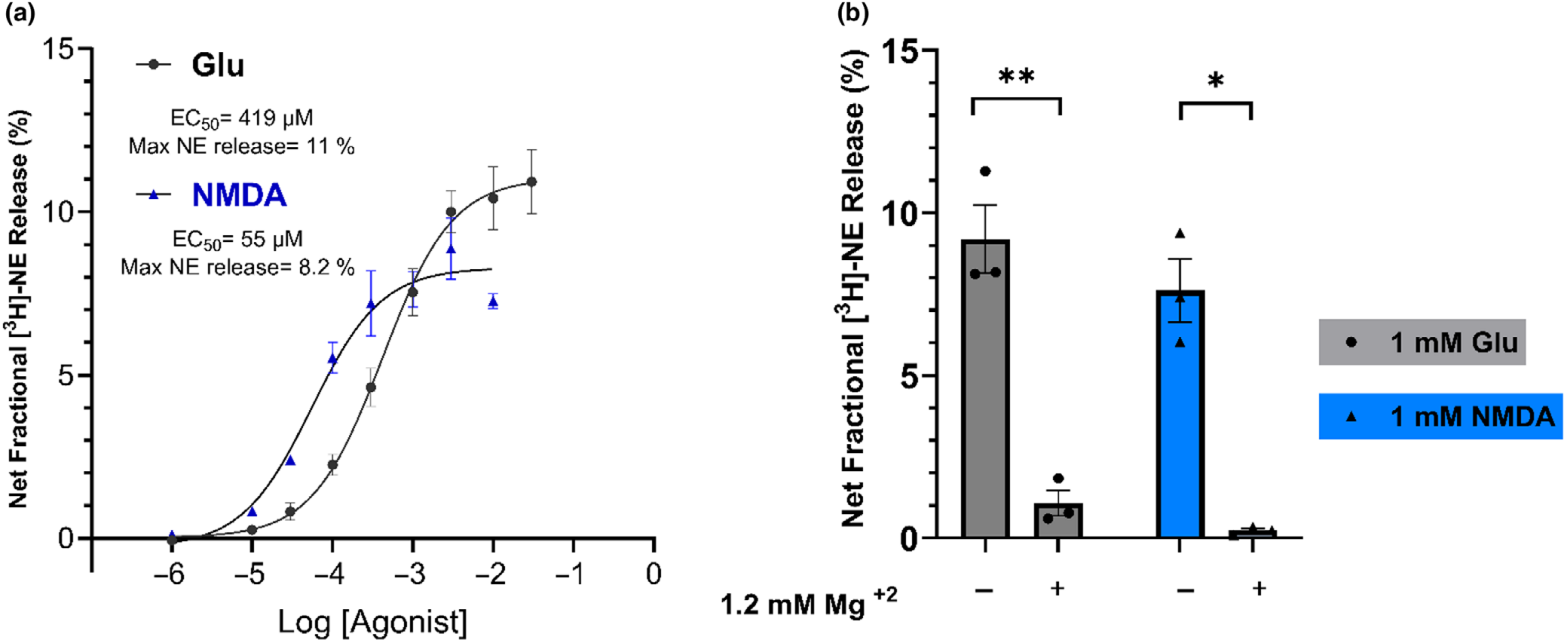
The stimulatory effect of Glutamate Vs. NMDA on [^3^H] ‐NE release in young rat cortical brain slices. In (a), the concentrations‐response curves of glutamate and NMDA‐stimulated [^3^H] ‐NE releases in the cerebral cortex tissue slices from young rats (*n* = 4). In (b), the 1 mM glutamate and NMDA stimulated NE release in the presence and absence of 1.2 mM magnesium in the cerebral cortex tissue slices from young rats (*n* = 3). Data were analyzed using an unpaired *t*‐test. *p ≤ 0.05, **p ≤ 0.01 NE, norepinephrine; Glu, glutamate; NMDA, N‐methyl‐d‐aspartate; Mg^2+^, magnesium.

To investigate the possible involvement of other ionotropic glutamate receptors in Glu‐stimulated [^3^H]‐NE release in the cortex, we directly compared the release stimulated by increasing concentrations of NMDA and glutamate. In cortical slices incubated in Mg^2+^‐free Krebs buffer, the net fractional [^3^H]‐NE release stimulated by glutamate and NMDA was concentration‐dependent, with EC_50_ values for glutamate of 419 μM [95% CL: 275 μM, 640 μM], and for NMDA of 55 μM [95% CL: 33 μM, 95 μM]. The maximum net fractional release of [^3^H]‐NE stimulated by glutamate was 11% [95% CL: 10.3 to 11.9%], and by NMDA it was 8.2% [95% CL: 7.6 to 9.0%] (Figure 4a). This difference in the concentration‐response curves indicates that NMDA acts as a potent partial agonist for [^3^H]‐NE release with ~75% efficacy compared to glutamate. The lower efficacy of NMDA compared to glutamate suggests that glutamate stimulates [^3^H]‐NE release predominantly but not exclusively via an NMDA receptor. To investigate this further, we measured release stimulated by 1 mM NMDA or glutamate in the absence or presence of an externally added physiological concentration of Mg^2+^ (1.2 mM) which is a voltage‐dependent blocker of the NMDA receptor. The added magnesium significantly blocked Glu‐stimulated NE release (~ 88%) with ~12% of the release still measurable (unpaired *t*‐test; *t* = 7.283, df = 4, *p* = 0.0019) indicating resistance to Mg^2+^ blockade. NMDA‐stimulated NE release was virtually abolished in the presence of magnesium (unpaired *t*‐test; *t* = 7.572, df = 4, *p* = 0.0016; Figure 4b). Together these results indicate that while Glu‐stimulated NE release in the rat cortex is mediated predominantly by NMDA receptors, a small but measurable fraction of Glu‐stimulated release might be mediated by a glutamate receptor that is not blocked by Mg^2+^, presumably a non‐NMDA glutamate receptor. This interpretation is consistent with previously reported results (Fink et al., 1992; Pittaluga & Raiteri, 1990).

### 3.5 Characterization of assembled NMDA receptors in the aged rat brain

**FIGURE 5:**
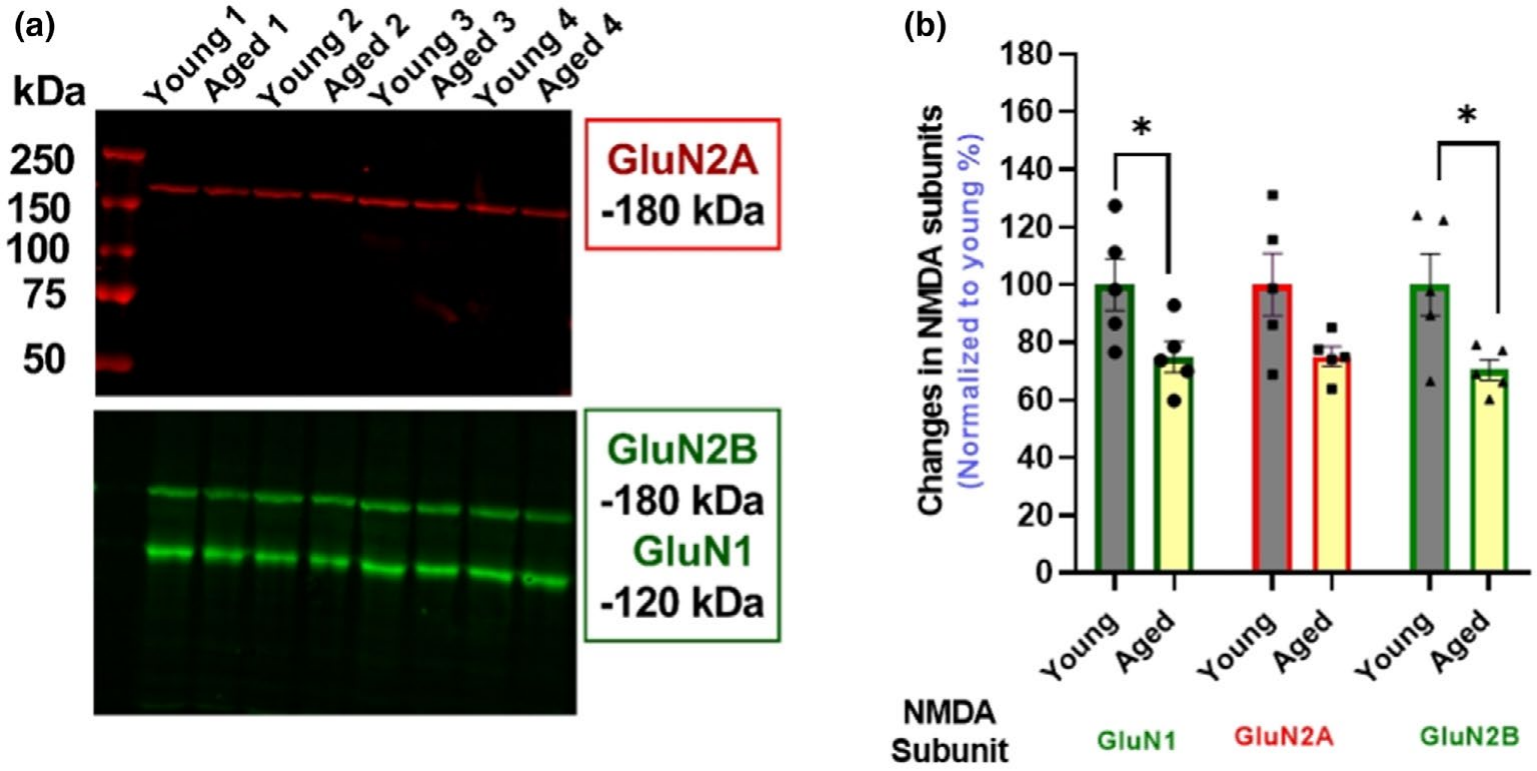
Age‐associated changes in the expression of NMDA receptors freely solubilize subunits in the cortical rat tissue homogenate. A representative Western blot for NMDA receptors subunits in the cerebral cortex in (a); and in (b) quantified results for GluN1, GluN2A, and GluN2B expressions in young and aged rats (*n* = 5). Data are expressed as mean (±SEM) normalized to young rats, with each data point representing a duplicate from one animal. Data were analyzed using an unpaired *t*‐test. *p ≤ 0.05. NMDA, N‐methyl‐d‐aspartate).

**FIGURE 6:**
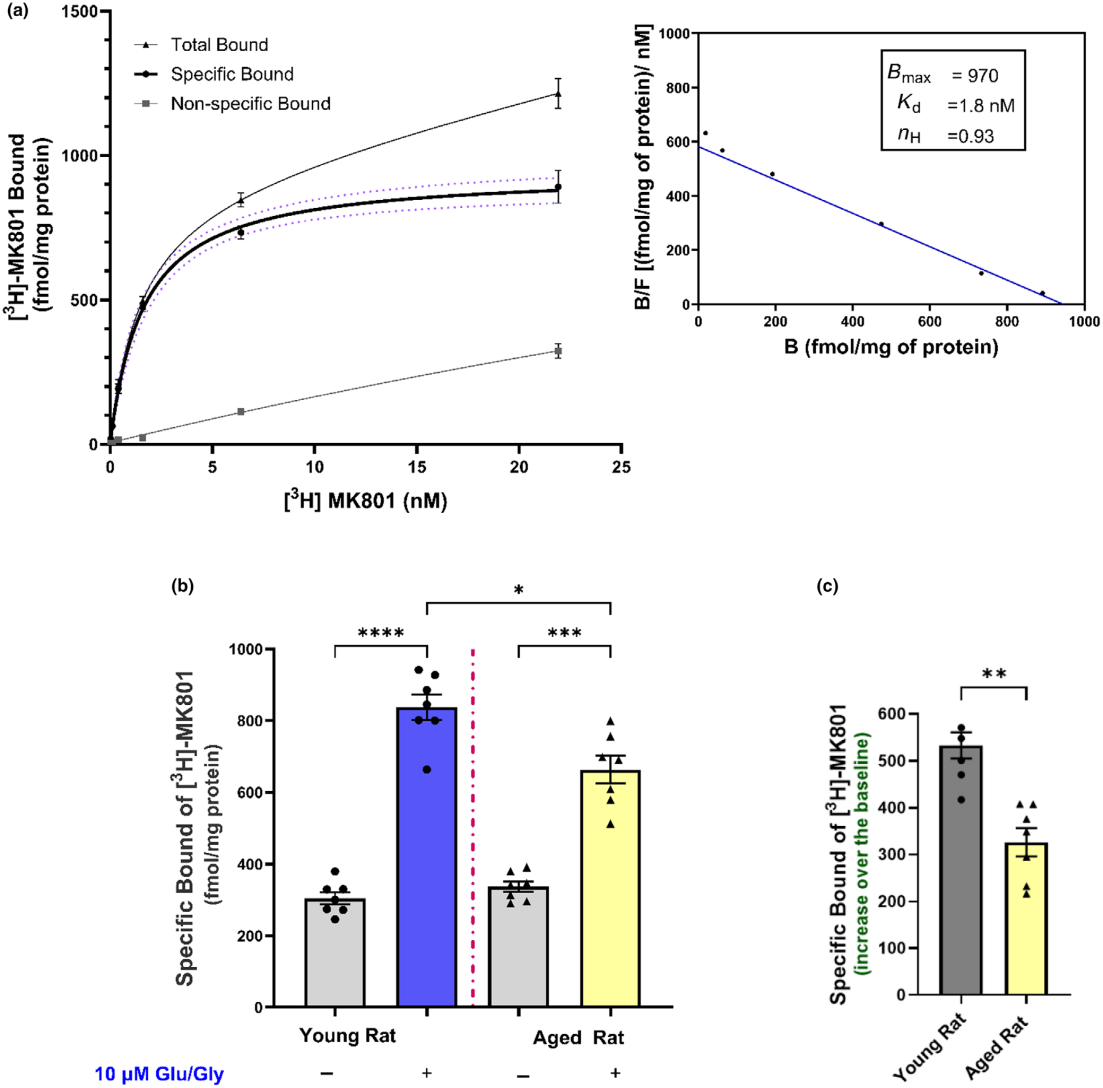
Effect of aging on [^3^H]‐MK‐801 binding to NMDA receptors in the young (2–3 months old) and aged (18–24 months old) rat cortical tissue membrane. In (a) saturation curve of [^3^H]‐MK‐801 binding to NMDA receptors in young rats in the presence of 10 μM Glu and Gly (*n* = 4). The non‐linear least‐squares fitting of the saturation isotherm yielded *K*_d_ and *B*_max_ values of 1.8 nM and 970 fmol/mg of protein, respectively. Both total and non‐specific binding of [^3^H]‐MK‐801 is shown in the curve, and the specific [^3^H]‐MK‐801 binding is presented with 95% CI in dotted lines. Inset: Saturation data graphed as Scatchard plots. Whereas in (b), the binding of 10 nM [^3^H]‐MK‐801 in +/− 10 μM Glu and Gly in young and aged rats (*n* = 7), each performed in triplicate and repeated twice. In (c), the % increases after subtracting baseline binding from the binding in the presence of 10 μM Glu and Gly. Baseline Binding values represent [^3^H]‐MK‐801 binding without exogenous addition of Glu and Gly. Data were analyzed using a Mixed‐effect analysis followed by Tukey's multiple comparison tests. *p ≤ 0.05, **p ≤ 0.01, ***p ≤ 0.001, ****p ≤ 0.0001; whereas the % of increase over the baseline was analyzed using an unpaired *t*‐test: **p ≤ 0.01. NMDA, N‐methyl‐d‐aspartate; Glu, Glutamate; Gly, Glycine; *K*_d_, dissociation constant; *B*_max_, Maximum Binding; n_H_, Hill Coefficient).

Our in vitro results showing that aging is associated with a significant decrease in Glu‐stimulated NE release but not release evoked by high potassium (Figure 1c) suggest that the release mechanism itself is not compromised. Instead, it points to an NMDA receptor‐mediated mechanism potentially underlying the age‐related decrease in Glu‐stimulated NE release in the rat cortex and hippocampus (Gonzales et al., 1991; Scognamiglio et al., 2024). Supporting this hypothesis, a deficit in NMDA receptors in the aged brain has been found in ligand binding studies (Magnusson, 1995, 2000; Piggott, Perry, Sahgal, & Perry, 1992), or inferred from Western blot assessments of the receptor subunits (Clayton & Browning, 2001; Kumar & Foster, 2019; Pegasiou et al., 2020; Zhao et al., 2009). To investigate whether the age‐related decrease in Glu‐stimulated release of NE could be explained by a change in the number of NMDA receptors or their response to glutamate and glycine, we first compared by Western blots the relative density of the NMDA receptor subunits GluN1, GluN2A and GluN2B in the cerebral cortex to determine if there were differences between young and aged rats. As shown in Figure 5, there is a statistically significant 25%–30% decrease in the GluN1 [unpaired *t*‐test; *t* = 2.394, df = 8, *p* = 0.0436] and GluN2B [unpaired *t*‐test; *t* = 2.619, df = 8, *p* = 0.0307] subunits respectively in the cortex from aged rats. However, any change in the levels of the GluN2A subunit did not reach statistical significance [unpaired *t*‐test; *t* = 2.182, df = 8, *p* = 0.0606]. These Western blot analyses are a measure of the NMDA receptor subunits present in the cortical whole homogenates, which would include assembled receptors, free subunits not yet assembled into receptors, and probably even subunits from degraded receptors. Therefore, we also investigated age‐related changes in NMDA receptors by measuring them in a ligand binding assay using [^3^H]‐MK‐801, which binds within the channel pore of NMDA receptors and thus presumably represents assembled receptors. We measured these [^3^H]‐MK‐801 binding sites in the presence of externally added 10 μM glutamate and 10 μM glycine, which increases the binding of the ligand, probably by facilitating access of the ligand to its binding sites in the channel pore (Foster & Wong, 1987; Serra, 1994; Song et al., 2018; Shah et al., 2023). As shown in Figure 6a, in well‐washed membranes in the presence of 10 μM added glutamate and glycine, [^3^H]‐MK‐801 binds to a high number of NMDA receptors in the rat cortex (*B*_max_ = 970 fmol/mg protein; 95% CL: 880–1110 fmol/mg) with high affinity (K_d_ = 1.8 nM; CL:1.3–2.8 nM). These binding saturation studies yielded Scatchard blots fit by a single straight line and a Hill coefficient (n_H_) of 0.93 (CL:0.74–1.16), consistent with a single population of binding sites (Figure 6a, inset). We next compared the number and regulation of NMDA receptors labeled by [^3^H]‐MK‐801 in well‐washed cortical membranes from young and aged rats in the absence and presence of added glutamate and glycine. In the absence of added glutamate and glycine, there was no difference in the density of NMDA receptors measured by the binding of 10 nM [^3^H]‐MK‐801, a concentration that is near the binding maximum, in cortical membranes from young and aged rats (Figure 6b). We then examined the effect of adding 10 μM glutamate and 10 μM glycine on the binding in cortical membranes from young and aged rats. In membranes from both young and aged rats the addition of 10 μM glutamate and glycine increased [^3^H]‐MK‐801 binding by 2‐fold or more, as analyzed by a mixed‐effects model [*F*_(1.946,11.68)_ = 149.9; *p* < 0.0001]. Importantly, the mixed‐effects model also revealed a significant effect of age; specifically, the addition of glutamate and glycine increased [^3^H]‐MK‐801 binding significantly more in the membranes from young rats than from aged rats [*F*_(6,18)_ = 4.338; *p* = 0.007; Figure 6b]. This difference in [^3^H]‐MK‐801 binding in the cortex from young and aged rats in the presence of glutamate and glycine is even clearer when expressed after subtracting the baseline binding measured in the absence of added glutamate (Figure 6c).

## 4 DISCUSSION

Glu‐stimulated release of NE in the cerebral cortex and hippocampus, which is mediated primarily by NMDA receptors, is significantly decreased in brain slices from aged rats (Gonzales et al., 1991; Pittaluga et al., 1993; Scognamiglio et al., 2024). In our previous studies, we have shown that this age‐associated deficit is rescued to the levels seen in young rats by the addition of d‐amphetamine (Scognamiglio et al., 2024), and in these series of studies, we show similar rescue with methylphenidate, another psychostimulant drug that shares certain pharmacological actions with d‐amphetamine, including inhibition of NE reuptake and possibly increased NE release (for review, see (Faraone, 2018; Sulzer et al., 2005)). The observed increase in Glu‐stimulated [^3^H]‐NE in the cortex in the presence of methylphenidate is interesting because the augmented released NE might be expected to actually limit its own release by feedback inhibition via α2‐adrenergic autoreceptors. Thus, the increased [^3^H]‐NE found indicates that the inhibition of NE reuptake by methylphenidate more than offsets any decrease in release due to feedback inhibition, resulting in a net increase in the measured extracellular NE. The same argument can be made for amphetamine, which also rescues the age‐related decrease in Glu‐stimulated [^3^H]NE release (Scognamiglio et al., 2024), although amphetamine has multiple mechanisms of actions in addition to inhibition of NE reuptake, including inhibition of vesicular storage, inhibition of monoamine oxidase, and reversal of the NE transporter (Faraone, 2018; Sulzer et al., 2005).

To further assess the scope of cellular mechanisms that could be targeted to augment NE release in the aging rat brain, we examined the potential role of inhibitory α2‐adrenergic receptors. These receptors function as inhibitory autoreceptors on NE axon terminals in the sympathetic nervous system (Langer et al., 1980; Starke, 2001; Weikop et al., 2004), as well as in NE axon projection areas of rat forebrain (Sacchetti et al., 2001). In both cases, when activated these receptors inhibit cyclic AMP production and lead to hyperpolarization of nerve terminals, thereby modulating NE release; thus, these receptors play an important role in regulating NE neurotransmission. Interestingly, a previous study found a decrease in α2‐adrenergic receptor binding sites in the prefrontal cortex from aged humans and from individuals who died with Alzheimer's disease, probably reflecting a loss of cortical NE axons (Kalaria & Andorn, 1991). This impaired NE release might reflect an inability to compensate for the loss of NE axons—i.e., a failure of homeostatic neurotransmission in the aging brain. In this regard, it is notable that reduced integrity of neurons in the locus coeruleus in the aging human brain (Mann, 1983) and in non‐human primates (Arnsten & Goldman‐Rakic, 1985) and the resulting decrease in NE neurotransmission compared with younger adults has been suggested to be a contributing factor in cognitive decline during aging (Arnsten & Goldman‐Rakic; Mann, 1983).

Our studies demonstrate that clonidine decreases the Glu‐stimulated release of NE indicating that inhibitory α2‐adrenergic receptors are, in fact, present in the cortical slices used here. Therefore, we determined if blocking these receptors was a reasonable and practical mechanism to restore NE release in the aging brain. We show here that drugs that block α2‐adrenergic receptors increase Glu‐stimulated NE release in brain slices from both young and aged rats and actually restore release in slices from aged rats to the level seen in slices from young rats in the absence of α2 blockers. Thus, blockade of these α2‐adrenergic receptors represents an alternative to the psychostimulants for augmenting synaptic NE release in the aging brain with implications for cognitive improvement. In fact, the literature has shown that various selective α2 blockers including idazoxan and mirtazapine have been shown to improve cognition in rats & mice (Chopin et al., 2004; Haapalinna et al., 2000; Nowakowska et al., 1999; Sara & Devauges, 1989; Singh et al., 2013) and humans with dementia (Coull et al., 1996).

The neuronal circuits through which glutamate stimulates NE release are not known, and we did not address this question directly, but our studies with the sodium channel blocker TTX, which blocks synaptic transmission, suggest that approximately 25% of the Glu‐stimulated NE release in the rat cortex is not dependent on TTX‐sensitive synaptic transmission; thus, this release may be mediated by NMDA receptors located directly on NE axon terminals or preterminals.

Together our results here, along with our previous characterization of Glu‐stimulated NE release (Scognamiglio et al., 2024) indicate that Glu‐stimulated NE release in the rat cortex is mediated predominantly by NMDA receptors, which are blocked by Mg^+2^ and sensitive to selective NMDA receptor orthosteric and ion channel blockers. However, our data do not rule out the possibility that a small fraction (perhaps 12%) of Glu‐stimulated release could be mediated by a glutamate receptor that is not blocked by Mg^+2^, presumably a non‐NMDA receptor. This interpretation would be consistent with previously reported studies in the rat hippocampus (Pittaluga & Raiteri, 1992).

An age‐related decrease in NMDA receptors has been found in the brains from rats, mice, non‐human primates, and humans (Pegasiou et al., 2020; Piggott, Perry, Perry, & Court, 1992; Wenk et al., 1991) for reviews, see (Magnusson, 1995; Newcomer et al., 2000). Consistent with a decrease in these receptors in the cortex from the aged rats studied here, our Western blot analyses indicated that there is a decrease in NMDA receptor subunits in the cerebral cortex from aged rats compared to young rats. In contrast, however, in the absence of added glutamate, which by activating the NMDA receptor opens the ion channel, we detected no decrease in the density of NMDA receptors measured by [^3^H]‐MK‐801 binding in well‐washed membrane homogenates. This difference in the results between the Western blot analyses of subunits and the ligand binding studies in these well‐washed membranes with no added glutamate and glycine (basal conditions) probably reflects what is measured by each method. The Western blots would have measured all NMDA receptor subunits present in the solubilized denatured homogenates, whether assembled into receptors or not, including nascent subunits not yet assembled into receptors and subunits present after receptor degradation, as well as the subunits actually assembled as NMDA receptors. In contrast, [^3^H]‐MK‐801, which binds mainly within the receptor channel, likely represents measurements of assembled receptors only. Interestingly, however, in contrast to the measurements of binding in the absence of glutamate and glycine (basal conditions), measurements of binding in the presence of added glutamate and glycine (activated conditions) reveal that there is, in fact, a marked decrease in the density of these activated NMDA receptors in the cortex from aged rats. This decrease is consistent with previous studies that found a decrease in [^3^H]‐MK‐801 binding in the brains of aged mice, rats, and humans in the presence of glutamate (Foster & Wong, 1987; Magnusson, 1995; Piggott, Perry, Sahgal, & Perry, 1992). Thus, a decrease in the glutamate‐activated NMDA receptor in the aged rat cortex could account for the decrease in Glu‐stimulated NE release.

An important question is why the marked decrease in [^3^H]‐MK‐801 binding in aged rat cortex is seen in the presence of glutamate but not in its absence. One possibility is that the NR2 subunit of cortical NMDA receptors may undergo a change between young adult and aged rat, as is thought to occur in the early stages of development (Monyer et al., 1994; Sheng et al., 1994; Watanabe et al., 1992). This could result in aged rat brain expressing predominantly receptors with faster deactivation kinetics, and/or faster desensitization, and shorter open time or probability of open receptor channels. If this were the case, the number of [^3^H]‐MK‐801 binding sites measured in or just before the channel entrance of inactive receptors in young and aged rat brain might be similar, but when activated by glutamate, the receptors from the aged brain would have a shorter open time compared to the receptors from young brain, and thus [^3^H]‐MK‐801 would have less access to the receptors located inside the channel lumen.

Alternatively, the NMDA receptors in aged rats may be less fluid or plastic than in younger rats and therefore not able to open as freely or as wide in the presence of glutamate as the receptor from young rats—i.e., an age‐related receptor fibrosis. This could account for decreased [^3^H]‐MK‐801 binding, as well as decreased receptor function, resulting in the decreased Glu‐stimulated NE release in aged brain.

Noradrenergic axons in the mammalian brain originate primarily from neurons in the locus coeruleus and project to nearly all brain regions, where the actions of NE is mediated by α‐ and β‐adrenergic receptors. Thus, NE participates in multiple and diverse CNS functions, including fundamental aspects of cognition such as arousal, attention, focus, and memory. Moreover, NE appears to play a direct role in memory formation by stimulating long‐term potentiation in hippocampal brain slices (Maity et al., 2015; Stanton & Sarvey, 1985), an effect mediated by β‐adrenergic receptors (reviewed in (O'Dell et al., 2015)). Consistent with NE acting via β‐adrenergic receptors in memory processes and performance in rats, the β‐adrenergic receptor antagonist propranolol blocks the amphetamine‐induced improvement of performance in the novel object recognition test (Scognamiglio et al., 2024). The importance of NE in cognition is reinforced by studies that found that a loss of NE neurotransmission in cerebral cortex is associated with cognitive decline in non‐human primates (reviewed in (Arnsten & Goldman‐Rakic, 1985) and in humans (Holland et al., 2021)).

NE release in the rat cortex is stimulated by glutamate, the primary excitatory neurotransmitter in the CNS, and modulated by inhibitory α‐2 adrenergic autoreceptors, which appear to be tonically active. Notably, glutamate can act synaptically, as well as by diffusing to extra‐synaptic glutamatergic receptors located on other elements, possibly including directly on noradrenergic terminals, where it might stimulate NE release. In either case, glutamate potently stimulates NE release from its axons (for review, see (Pittaluga, 2021)). During the aging process, the glutamatergic system becomes dysfunctional, leading to changes in release and reuptake mechanisms for glutamate itself (Segovia et al., 2001; Zahr et al., 2008). The NMDA receptors in the cortex are crucial for, among other things, memory formation and for their role in regulating long‐term potentiation (Bliss & Collingridge, 1993; Rison & Stanton, 1995); thus, changes in these receptors during aging may have an important influence on learning and memory (Bliss & Collingridge, 1993; Rison & Stanton, 1995).

Ta ken together, our studies here demonstrate that Glu‐stimulated NE release, which is mediated primarily by NMDA receptors, can be augmented by the psychostimulant methylphenidate, as well as by blocking the inhibitory α2‐adrenoreceptors with drugs like mirtazapine and idazoxan. These results have clinical implications, since augmented NE release may represent an important pharmacological strategy to restore neurotransmitter imbalances in the aging brain and some aspects of cognition in the aging population.

## AUTHOR CONTRIBUTIONS

**Yousef Aljohani:** Methodology; writing – review and editing; validation; conceptualization; writing – original draft. **William Payne:** Methodology. **Robert P. Yasuda:** Methodology; validation. **Thao Olson:** Methodology; validation. **Kenneth J. Kellar:** Methodology; conceptualization; validation; writing – review and editing; writing – original draft. **Ghazaul Dezfuli:** Conceptualization; methodology; validation; writing – review and editing; writing – original draft.

## CONFLICT OF INTEREST STATEMENT

The authors declare that the research was directed without commercial or financial relationships that could be interpreted as a potential conflict of interest.

### PEER REVIEW

The peer review history for this article is available at https://www.webofscience.com/api/gateway/wos/peer‐review/10.1111/jnc.16280.

## CLASSIFICATION

Paste the major and minor classifications here. Dual classifications are permitted but cannot be within the same major classification.

## Data Availability

The data that support the findings of this study, and any additional information are available upon request from the lead contact Ghazaul Dezfuli (gd96@georgetown.edu).
